# Impact of defacing on automated brain atrophy estimation

**DOI:** 10.1186/s13244-022-01195-7

**Published:** 2022-03-26

**Authors:** Christian Rubbert, Luisa Wolf, Bernd Turowski, Dennis M. Hedderich, Christian Gaser, Robert Dahnke, Julian Caspers, for the Alzheimer’s Disease Neuroimaging Initiative

**Affiliations:** 1 University Dusseldorf, Medical Faculty, Department of Diagnostic and Interventional Radiology, D-40225 Dusseldorf, Germany; 2 Department of Diagnostic and Interventional Neuroradiology, School of Medicine, Technical University of Munich, 81675 Munich, Germany; 3 Departments of Neurology and Psychiatry, Jena University Hospital, 07745 Jena, Germany; 4 Institut of Psychology, Friedrich Schiller University Jena, 07743 Jena, Germany; 5 Center of Functionally Integrative Neuroscience, Aarhus University, 8000 Aarhus, Denmark

**Keywords:** Magnetic resonance imaging, Brain, Atrophy, De-identification, Privacy

## Abstract

**Background:**

Defacing has become mandatory for anonymization of brain MRI scans; however, concerns regarding data integrity were raised. Thus, we systematically evaluated the effect of different defacing procedures on automated brain atrophy estimation.

**Methods:**

In total, 268 Alzheimer’s disease patients were included from ADNI, which included unaccelerated (*n* = 154), within-session unaccelerated repeat (*n* = 67) and accelerated 3D T1 imaging (*n* = 114).

Atrophy maps were computed using the open-source software veganbagel for every original, unmodified scan and after defacing using afni_refacer, fsl_deface, mri_deface, mri_reface, PyDeface or spm_deface, and the root-mean-square error (RMSE) between z-scores was calculated.

RMSE values derived from unaccelerated and unaccelerated repeat imaging served as a benchmark. Outliers were defined as RMSE > 75th percentile and by using Grubbs’s test.

**Results:**

Benchmark RMSE was 0.28 ± 0.1 (range 0.12–0.58, 75th percentile 0.33).

Outliers were found for unaccelerated and accelerated T1 imaging using the 75th percentile cutoff: afni_refacer (unaccelerated: 18, accelerated: 16), fsl_deface (unaccelerated: 4, accelerated: 18), mri_deface (unaccelerated: 0, accelerated: 15), mri_reface (unaccelerated: 0, accelerated: 2) and spm_deface (unaccelerated: 0, accelerated: 7). PyDeface performed best with no outliers (unaccelerated mean RMSE 0.08 ± 0.05, accelerated mean RMSE 0.07 ± 0.05).

The following outliers were found according to Grubbs’s test: afni_refacer (unaccelerated: 16, accelerated: 13), fsl_deface (unaccelerated: 10, accelerated: 21), mri_deface (unaccelerated: 7, accelerated: 20), mri_reface (unaccelerated: 7, accelerated: 6), PyDeface (unaccelerated: 5, accelerated: 8) and spm_deface (unaccelerated: 10, accelerated: 12).

**Conclusion:**

Most defacing approaches have an impact on atrophy estimation, especially in accelerated 3D T1 imaging. Only PyDeface showed good results with negligible impact on atrophy estimation.

## Key points

- Background: defacing MRI examinations of the brain is important to preserve privacy.
- Defacing procedures may interfere with software-based brain atrophy estimation (e.g., veganbagel).
- Most defacing procedures lead to systematic bias concerning atrophy estimation.
- Substantial z-score deviations were found in 0–17.9% compared to a benchmark.
- Only defacing with PyDeface had a negligible impact on atrophy estimation.

## Background

Magnetic resonance imaging (MRI) studies of the brain usually include facial features of a patient. For example, brain imaging in patients suffering from a neurocognitive decline or neurodegenerative diseases usually include a 3D T1-weighted anatomical dataset, which commonly depicts the ears and face. The threat of identifying patients or research subjects by applying face recognition techniques to such MRIs has been increasingly recognized in recent years. While only 40% of human volunteers were able to match face reconstructions based on volumetric renderings of 3D MRI data to the respective participants photographs with a success rate of “greater than chance” in a study from 2009 [1], studies employing machine learning approaches were successful in 83% of the cases in 2019 [2] and in 97% of the cases in 2021 [3].

Therefore, it is highly desirable—and frankly necessary—to remove identifying features, such as the face or ears [1–4], from MRI examinations depicting these in detail prior to a public data release or even before submitting MRI scans to an off-site service, e.g., to a cloud-based service for brain atrophy estimation. Several defacing approaches have been developed, some of which were applied to large-scale cohort studies, such as the Human Connectome Project (HCP [5]) and the Nathan Kline Institute—Rockland Sample (Rockland [6]). The most popular defacing approaches include afni_refacer (based on AFNI [7]), mask_face (released by the Neuroinformatics Research Group [8]), mri_deface (based on FreeSurfer [9]), fsl_deface (based on FMRIB’s Software Library (FSL) [10]), PyDeface (released by the Poldrack Lab [11]) and spm_deface (included in the Statistical Parametric Mapping (SPM) package [12]). However, concerns have been raised with respect to data alteration after defacing, with some studies reporting significant deviations in brain volume assessments [13, 14], while other studies have shown almost no effects of defacing [3, 15].

Brain atrophy is a feature of many neurodegenerative diseases, and characteristic brain volume changes may be decisive for diagnosis, for example in Alzheimer’s disease (AD [16]) or frontotemporal dementia [17], among others [18–20]. Brain volume changes are furthermore increasingly used in treatment monitoring, for example in the early stages of multiple sclerosis. [21] Several software packages for evaluation of regional brain volume alterations have been made available in the recent years. Software-augmented reading has been shown to help detect subtle volume losses in the early course of a disease and to decrease high inter-reader variation in reporting of regional brain atrophy [22, 23].

While technical accuracy is a hallmark of volumetric brain atrophy estimation, the impact of defacing procedures on the result has not been systematically studied. The current study evaluates the impact of commonly used defacing procedures on brain atrophy z-score maps in a large sample from the ADNI cohort using veganbagel, an open-source software for automatic brain atrophy estimation built around CAT12 for SPM12.

## Methods

AD patients from the Alzheimer’s Disease Neuroimaging Initiative (ADNI [24]) database were retrospectively included in the analysis. The ADNI was launched in 2003 as a public–private partnership, led by Principal Investigator Michael W. Weiner, MD (http://www.adni-info.org/). AD patients were included, when (1) there was a 3D T1-weighted MRI series acquired at the Screening visit with a slice thickness of ≤ 1.5 mm, and (2) patients were aged younger than 75 years at the time of the MRI. Any MRI acquisition failing ADNI’s quality control (QC) were excluded. Unaccelerated repeats passing QC were excluded, when the initial imaging failed QC. A total of 268 AD patients were included (Table 1). The study was approved by the local ethics committee. Only publicly available data were used. Statistical analysis was carried out using R [25].

**Table 1:** Demographics of all included patients from the Alzheimer’s Disease Neuroimaging Initiative (ADNI) as well as the analyzed subgroups

	*n*	Females	Age	Study phase (ADNI 1/2/3)	Scanners^a^	GE/Philips/Siemens (1.5 T/3 T)
All AD patients	268	141 (52.6%)	67.7 ± 5.3 (55–74)	29%/56%/15%	95	38%/19%/43% (29%/71%)
Unaccelerated imaging	154	82 (53.2%)	67.8 ± 5.1 (55–74)	51%/49%/0%	76	45%/16%/38% (51%/49%)
Unaccelerated repeat imaging	67	38 (56.7%)	68.1 ± 5.0 (56–74)	100%/0%/0%	38	58%/6%/36% (100%/0%)
Accelerated imaging	114	59 (51.8%)	67.6 ± 5.5 (55–74)	0%/65%/35%	56	28%/24%/48% (0%/100%)

GE, general electric (Boston, MA, USA); Philips, Koninklijke Philips (Amsterdam, the Netherlands); Siemens, Siemens Healthineers (Erlangen, Germany); T, Tesla

^a^The number of scanners is estimated from the scanner device serial number included in the DICOM headers

In the ADNI, 3D T1 images were acquired (1) as unaccelerated imaging, (2) as unaccelerated repeat imaging (repetition of an unaccelerated 3D T1 within the same session) or (3) as accelerated imaging (i.e., parallel imaging using GRAPPA [26], SENSE [27] or the closely related ASSET). All patients with unaccelerated repeat imaging were also part of the group with unaccelerated imaging. Studies with different approaches to atrophy estimation have shown varying degrees of differences in atrophy assessments when using accelerated vs. unaccelerated imaging [28–31]. Since potentially different effects of defacing on accelerated vs. unaccelerated imaging have so far not been systematically studied, we show results for unaccelerated and accelerated 3D T1 imaging separately.

The previously published open-source software for volumetric estimation of gross atrophy and brain age longitudinally (veganbagel), an automatic workflow for generation of atrophy maps relative to age- and sex-specific normal templates [32], was adapted for the analysis. The latest available Docker-based version of veganbagel was used, containing the standalone version of CAT12.7 without the need for a separate MATLAB-license (https://github.com/BrainImAccs/veganbagel, commit 6a2ac5f). veganbagel was chosen for the analysis, since it is based on the established CAT12 for SPM12 software package and, to our knowledge, is the only open-source software readily allowing for single time point atrophy estimations for an individual brain scan.

In the workflow, standardized preprocessing of structural T1-weighted imaging is performed, comprising gray matter segmentation, normalization, modulation and spatial smoothing using CAT12 for SPM12. After preprocessing of healthy reference subjects from a normal cohort (Rockland [6], “Baseline1” visits, *n* = 949 (65% female), mean age = 46.3 ± 17.1 years (range 18–77)), mean and standard deviation (SD) templates are generated for each sex and age (containing the actual age  ± 2 years).

Z-score maps (= “atrophy maps”) were then calculated for all AD patients using the equally preprocessed, unmodified, full face 3D T1 series (= “full face”) as well as algorithmically defaced 3D T1 series using the aforementioned age- and sex-specific templates. Six defacing approaches were applied separately: afni_refacer (AFNI v21.0.21 [7]), fsl_deface (FSL v6.0.3 [10]), mri_deface (FreeSurfer v7.1.1 [9]), mri_reface v0.2 [3], PyDeface v2.0.0 from the Poldrack Lab [11] and spm_deface from SPM12 r7771 [12]. The previously mentioned mask_face (released by the Neuroinformatics Research Group [8]) was excluded from the analysis, since the defacing has been shown to be reversible using Cycle-Consistent Adversarial Networks [33]. mri_deface, fsl_deface and PyDeface each apply a linear registration, atlas and mask-based approach to identify the face and remove it. fsl_deface also removes the ears. afni_refacer and mri_reface replace the ears and face with a population average. mri_reface furthermore replaces some regions of air, which may include identifiable features due to wraparound artifacts. All defacing approaches were run with their respective default settings. The automatic generation of QC images, offered by afni_refacer and mri_reface, was disabled.

To analyze the impact of different defacing approaches on veganbagel’s atrophy estimation, gray matter atrophy z-scores after defacing were compared to the respective z-scores derived from the unmodified full face data. Specifically, for each voxel within the gray matter mask used in the veganbagel workflow, the root-mean-square error (RMSE) was calculated for the difference of the z-score after defacing minus the respective full face z-score.

Grubbs’s test, also known as the extreme studentized deviate test or maximum normalized residual test, was performed to identify outliers within the RMSE values of each defacing approach. Grubbs’s test by design only detects a single outlier. Therefore, the test was performed iteratively, i.e., the detected outlier was removed before rerunning the test until no more outliers are detected. In brief, Grubbs’s test is based on the difference of the mean and the minimum or maximum value of the data as determined by the standard deviation [34]. Grubbs’s test may produce false positives in distributions with a very large or very small standard deviation [35]. Therefore, in order to provide further context for the number of outliers detected by Grubbs’s test and to serve as a benchmark, z-score maps based on unmodified full face, unaccelerated 3D T1 imaging were compared to unmodified full face, unaccelerated within-session repeat 3D T1 imaging of the same subject, if available from the ADNI database (see Table 1, no repeats of accelerated imaging were acquired during ADNI). For each defacing approach the number of outliers with respect to the 75th percentile of the RMSE values of the benchmark results are reported.

Lastly, to visualize the regions most affected by each defacing approach, the absolute mean differences of the z-scores were plotted as a heat map onto representative axial slices of the SPM152 standard template, masked by the same, using MRIcroGL [36].

## Results

**Fig. 1:**
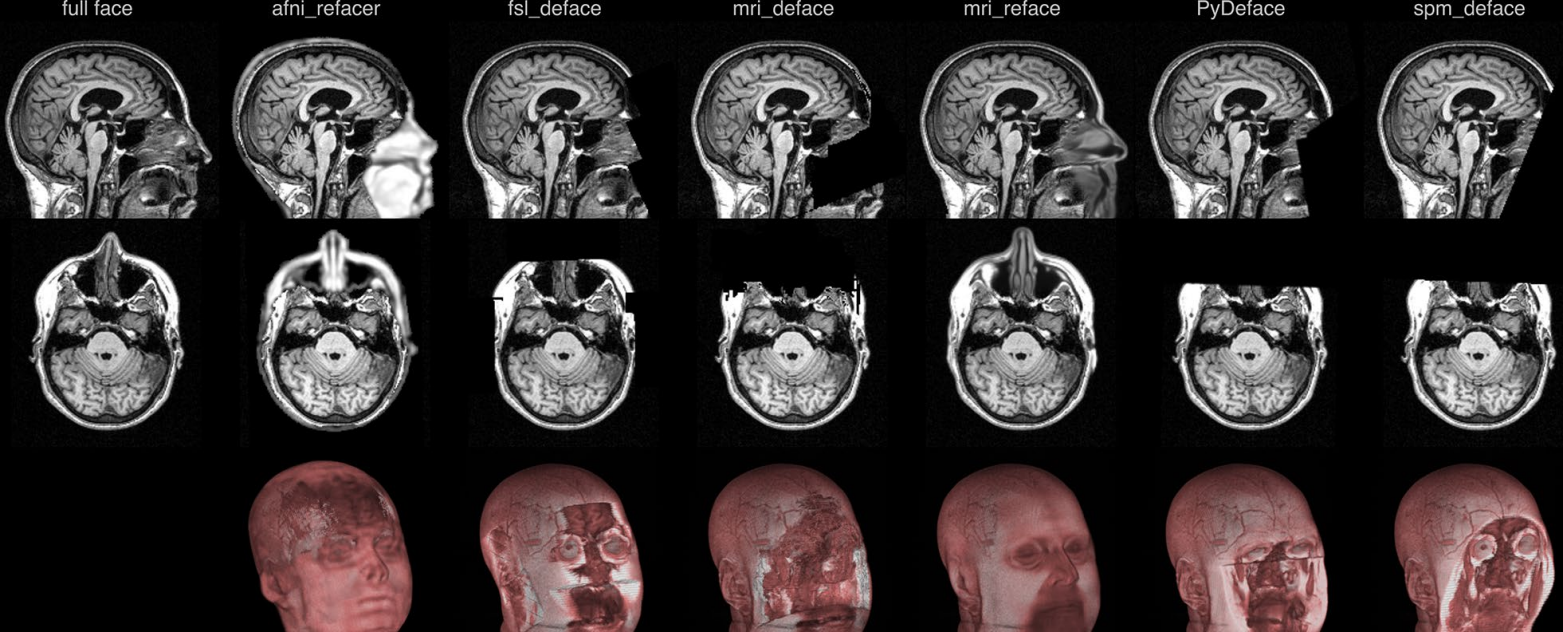
Example of successful defacing approaches on an Alzheimer’s disease patient (female, 69 years of age). Top row shows the sagittal reformations of the defaced image volume, second row shows the axial reformations and the bottom row shows volume renderings. The volume rendering of the full face image (lower left) has been omitted for privacy reasons

In a total of 1877 of 1943 attempts (96.6%), the combination of defacing and the veganbagel workflow completed successfully. Examples are shown in Fig. 1.

afni_refacer failed in two unaccelerated 3D T1 imaging, not yielding imaging volumes usable for further processing. mri_deface crashed while processing 32 unaccelerated and 28 accelerated 3D T1 scans. In two accelerated 3D T1 imaging acquisitions, mri_deface completed, but the adaptive maximum a posterior (AMAP)-based segmentation step in CAT12 detected untypical tissue peaks and stopped further processing. The latter also occurred in two accelerated imaging acquisitions when using spm_deface. In all other approaches, including full face and unaccelerated repeat imaging, no failed processing was noted.

**Fig. 2:**
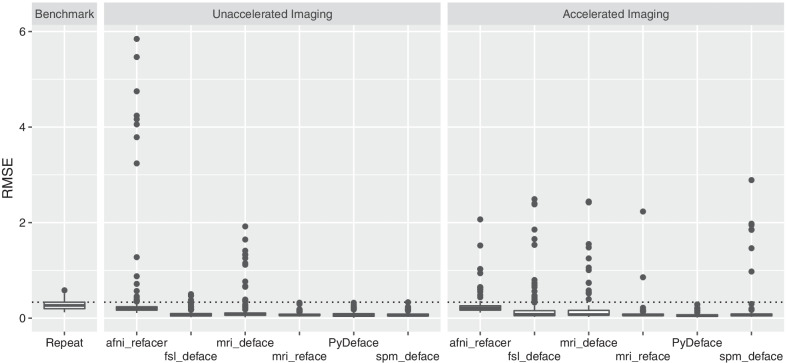
Box-and-whisker plots of the root-mean-square error (RMSE) values after veganbagel processing of Alzheimer’s disease patients. Left column: The benchmark result obtained by calculating the RMSE between gray matter z-scores of full face unaccelerated 3D T1 imaging and the respective same-session unaccelerated repeat 3D T1 imaging. Center and right: RMSE values obtained by comparing z-scores of defaced unaccelerated (center) or accelerated (right) 3D T1 imaging series with the respective full face 3D T1 imaging. The dotted black line denotes the 75th percentile of the RMSE values of the benchmark

The RMSE for the benchmark, comparing the gray matter z-scores of the full face unaccelerated 3D T1 imaging with the respective unaccelerated repeat imaging, is shown in the left column of Fig. 2. The mean benchmark RMSE was 0.28 ± 0.1 (minimum: 0.12, 75th percentile: 0.33 and maximum: 0.58). No outliers were detected in the benchmark RMSE values using Grubbs’s test.

The RMSE comparing the gray matter z-scores based on the defaced 3D T1 imaging with the full face 3D T1 imaging series are shown in the center (unaccelerated imaging) and right (accelerated imaging) column of Fig. 2, respectively. RMSE values and outliers are furthermore reported in Table 2.

**Table 2:** Voxel-wise z-score root-mean-square error (RMSE) values after veganbagel processing of Alzheimer’s disease patients and comparing the results after defacing with the respective z-scores derived from the respective full face 3D T1 imaging

	Unaccelerated imaging					Accelerated imaging				
	Failed processing^a^	Mean RMSE ± SD	Range (IQR)	Outliers		Failed processing^a^	Mean RMSE ± SD	Range (IQR)	Outliers	
				Grubbs’s test	Benchmark^b^				Grubbs’s test	Benchmark^b^
afni_refacer	2/154	0.45 ± 0.97	0.11–5.84 (0.24–2.16)	16 (10.5%)	18 (11.8%)	0/114	0.28 ± 0.26	0.12–2.07 (0.26–0.65)	13 (11.4%)	16 (14%)
fsl_deface	0/154	0.09 ± 0.08	0.02–0.50 (0.10–0.24)	10 (6.5%)	4 (2.6%)	0/114	0.23 ± 0.47	0.03–2.49 (0.16–1.06)	21 (18.4%)	18 (15.8%)
mri_deface	32/154	0.20 ± 0.35	0.02–1.92 (0.11–1.15)	25 (20.5%)	15 (12.3%)	30/114	0.30 ± 0.57	0.03–2.44 (0.16–1.54)	20 (23.8%)	15 (17.9%)
mri_reface	0/154	0.08 ± 0.04	0.03–0.32 (0.08–0.13)	7 (4.5%)	0 (0%)	0/114	0.10 ± 0.22	0.04–2.23 (0.09–0.14)	6 (5.3%)	2 (1.8%)
PyDeface	0/154	0.08 ± 0.05	0.01–0.32 (0.09–0.19)	5 (3.2%)	0 (0%)	0/114	0.07 ± 0.05	0.01–0.29 (0.07–0.17)	8 (7%)	0 (0%)
spm_deface	0/154	0.07 ± 0.05	0.03–0.33 (0.09–0.18)	10 (6.5%)	0 (0%)	2/114	0.18 ± 0.45	0.03–2.89 (0.09–1.20)	12 (10.5%)	7 (6.1%)

SD, standard deviation; IQR, interquartile range

^a^Failed processing denotes defacing crashing (the majority of cases) or veganbagel (i.e., CAT12 for SPM12) processing failing on the defaced image volume (*n* = 2 for mri_deface and *n* = 2 for spm_deface). Outliers are reported using Grubbs’s test for each individual approach

^b^By counting any RMSE values which were higher than the 75th percentile of the RMSE values of the benchmark result (0.33)

For defacing unaccelerated 3D T1 imaging, excellent results with a very small RMSE were found when applying fsl_deface, mri_reface, PyDeface and spm_deface. However, Grubbs’s test detected 10, 7, 5 and 10 outliers, respectively, and for fsl_deface, 4 outliers were detected with respect to the 75^th^ percentile of the RMSE values of the benchmark. Using afni_refacer leads to a higher mean RMSE in comparison with the other approaches and 16 outliers according to Grubbs’s test and 18 outliers with respect to the benchmark were noted, respectively. mri_deface, in comparison, leads to overall smaller RMSE values, but also results in several outliers (25 and 15, respectively) and, as noted above, crashed in a substantial amount of cases.

For the accelerated 3D T1 imaging, the smallest mean RMSE was obtained using PyDeface with 8 outliers in Grubbs’s test, which were found within a small range of the RMSE values from 0.01 to 0.29, and no outliers in comparison with the 75th percentile of the RMSE values of the benchmark. mri_reface showed very good results overall with a small mean RMSE and a very small IQR. Six outliers were found according to Grubbs’s test, but also two outliers were noted in comparison with the benchmark with a RMSE of 0.86 and a relatively high RMSE of 2.23. Reviewing the defaced imaging volumes, no obvious errors in the defacing process were noted. spm_deface and fsl_deface performed worse in comparison with the unaccelerated 3D T1 imaging, with a higher mean RMSE and several more outliers. mri_deface shows an overall similar performance when compared to the results from unaccelerated 3D T1 imaging with a slightly higher mean RMSE and wider range, as well as a comparable number of crashes. afni_refacer results were better when compared to the unaccelerated imaging results, but still a comparable number of outliers were noted.

**Fig. 3:**
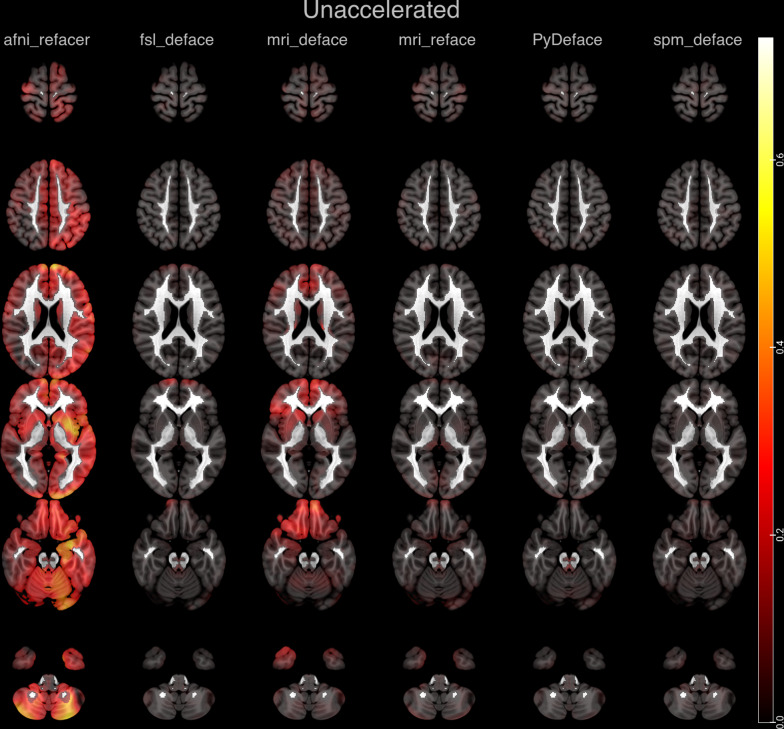
Absolute mean differences of the z-scores plotted as a heat map onto representative axial slices of the SPM152 standard template after applying the different defacing approaches on unaccelerated imaging

**Fig. 4:**
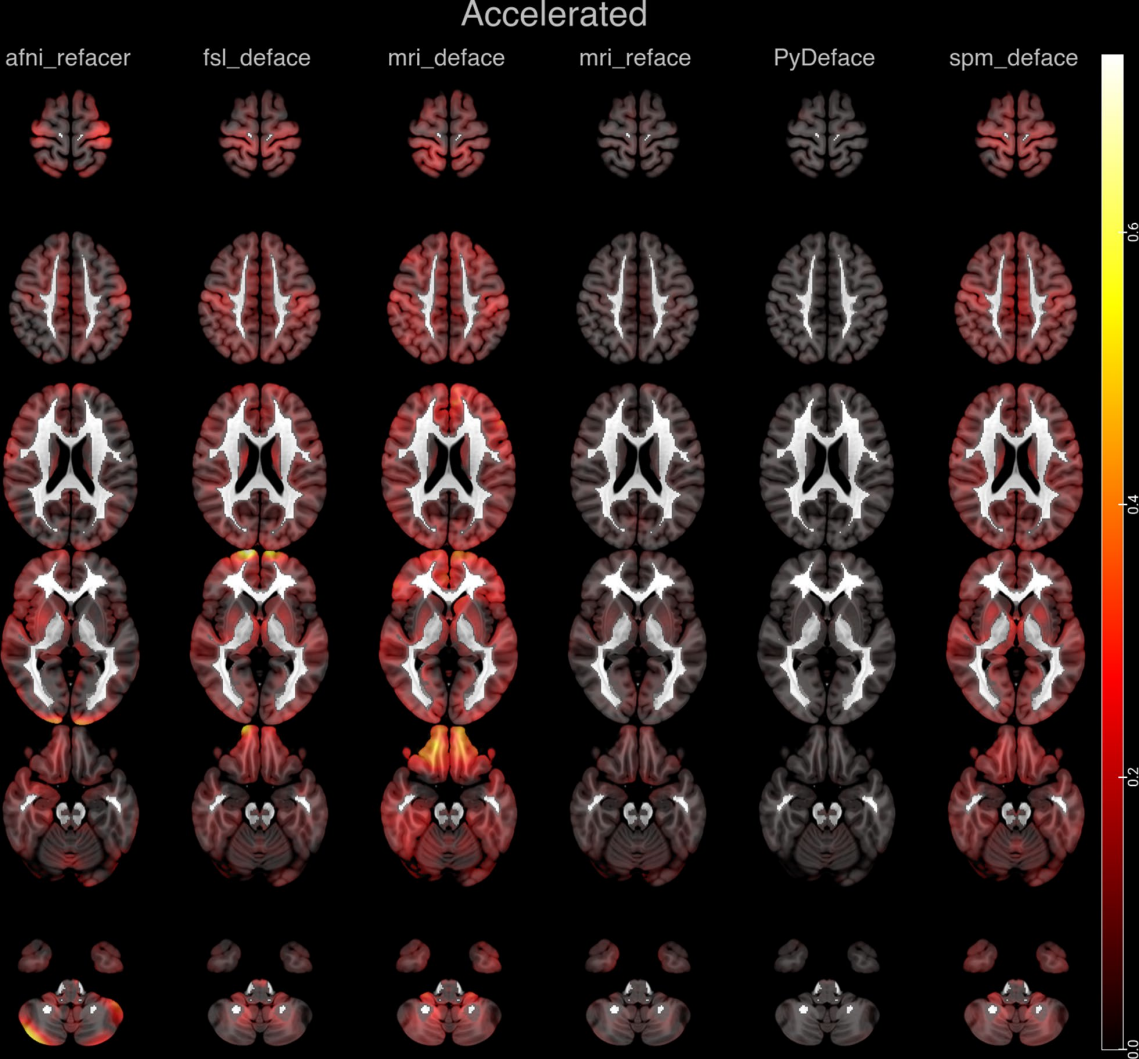
Absolute mean differences of the z-scores plotted as a heat map onto representative axial slices of the SPM152 standard template after applying the different defacing approaches on accelerated imaging

In order to visualize the regions most affected by each defacing approach, heat maps for the absolute mean differences of the z-scores between defaced and unmodified full face z-score maps are shown in Fig. 3 for unaccelerated and in Fig. 4 for accelerated imaging.

In unaccelerated imaging, afni_refacer shows marked global deviations in z-scores, predominately over the left hemisphere. mri_deface shows marked differences in the frontobasal brain and temporal poles, including changes in the basal ganglia, especially the caudate nuclei. fsl_deface shows small focal differences in the frontal and frontobasal brain. The differences after mri_reface, PyDeface and spm_deface are less pronounced, with very small deviations, e.g., in the anterior frontobasal cortex, the thalami and occipital cortex after mri_reface.

In accelerated imaging, in line with the previous analyses of the RMSE, the mean differences are globally higher after fsl_deface, mri_deface and spm_deface. fsl_deface and mri_deface furthermore follow the same pattern of deviations with more pronounced deviations in the frontal (fsl_deface) and frontobasal brain (fsl_deface, mri_deface). spm_deface shows globally increased mean differences, especially including the basal ganglia. mri_reface also shows a very slight global increase in mean differences, in accelerated imaging with a slight emphasis on the putamen, and less on the thalami. After afni_refacer, less absolute mean differences overall are noted in accelerated imaging in comparison with unaccelerated imaging, showing a predominance over the right hemisphere and focal differences along the occipital cortex and superficial cerebellum.

PyDeface shows no discernible patterns for both unaccelerated and accelerated imaging.

## Discussion

In the present study, we evaluated the impact of different defacing approaches on veganbagel, an open-source software built around CAT12 for SPM12. veganbagel allows for automatic brain atrophy estimation by comparing a subject’s structural brain scan to a normal cohort. Our results indicate that most defacing procedures are robust, with the exception of mri_deface. Most of the defacing approaches introduced pronounced z-score deviations in the context of automatic brain atrophy estimation. The smallest bias with no notable outliers in comparison with the benchmark results was found when using PyDeface.

In recent years, ever stricter laws and regulations on data collection and processing have been imposed, introducing severe penalties for mishandling of information and data breaches. Recently, machine learning-based face recognition approaches have been shown to be alarmingly successful in matching photographs of participants to their respective MRI scans, with a success rate of up to 97% [2, 3]. On the other hand, concerns have been raised on the data integrity after defacing, especially with regard to common volumetric analysis [13, 14], while other studies have shown modest to no effects of defacing on common neuroscientific analysis pipelines [3, 15].

Currently, there are two main approaches for anonymizing brain imaging data, “skull stripping,” i.e., removing everything from the image volume except for the brain, and removing just the facial features from the imaging volume, while retaining all other information. Automated skull stripping does suffer from limitations [37], and (accidental) removal of some voxels of the brain might severely limit any downstream analysis. Therefore, defacing is often the preferred method, which is supposed to retain all information of the brain. The approaches tested in the current study generally register a brain scan to a common template and then apply a mask-based detection of the facial features to either remove these areas or replace the respective areas with a separately derived population average. In theory, defacing should therefore not interfere with, e.g., automated atrophy detection. However, we have noted deviations in a number of cases with sometimes large RMSEs, with no obvious changes to the brain in the defaced image volumes. Some neuroscientific analysis pipelines, such as CAT12/SPM12, may use information from the background of the image volume in the detection of tissue classes (gray and white matter, cerebrospinal fluid), which might explain the observed deviations after defacing.

veganbagel denotes z-scores between − 2.5 to 2.5 as volume changes within expected limits [32]. With respect to these we found an extremely small mean RMSE in the benchmark assessments of full face unaccelerated 3D T1 imaging and the respective unaccelerated repeats. veganbagel may therefore be considered to yield robust results in a heterogeneous dataset acquired on multiple different scanners.

We found that atrophy estimation results were susceptible to changes when defacing is conducted using afni_refacer, fsl_deface (pronounced in accelerated 3D T1 imaging), mri_deface or spm_deface (only in accelerated 3D T1 imaging), but not PyDeface. PyDeface resulted in a very small mean RMSE, no outliers noted with respect to the benchmark results and no clearly discernible pattern of deviations in the absolute mean differences maps. There were some outliers found by Grubbs’s test, but these must be placed in the context of a very small range of RMSE values, smaller, in fact, than the range of the benchmark RMSE values. In essence, no relevant changes in atrophy estimation are expected when processing brain scans defaced using PyDeface with veganbagel. Visualizing the regions of the brain most affected after defacing, fsl_deface and mri_deface demonstrated deviations mostly close to the face, but in accelerated imaging, more global deviations were observed for fsl_deface, mri_deface and spm_deface, including the cerebellum and basal ganglia. Last, but not least, it has to be noted that in two cases both afni_refacer and spm_deface failed to yield image volumes usable for further analysis, while mri_deface was not able to process a substantial number of the brain scans with the default settings. mri_reface performed slightly better in this regard and showed very good overall results in our current study with regard to the RMSE, but two outliers, one of which with a relatively high RMSE, were noted after defacing accelerated 3D T1 imaging acquisitions.

Further considering privacy issues, mri_deface and PyDeface sometimes retain parts of the orbits after defacing [3], and even imperfect, partial data may be of use for face recognition. [38] Schwarz et al. have shown that machine learning-based face recognition is still successful in 3% (fsl_deface) to 10% (mri_deface and PyDeface) of the cases after defacing by removing identifying parts of the face. However, the success rate of identifying subjects after defacing may be pushed to 28–38% when replacing missing parts of the face in defaced image volumes with a population average (fsl_deface = 28%, mri_reface = 30%, mri_deface = 33% and PyDeface = 38%). [3]

Furthermore, we have noted differences after defacing unaccelerated and accelerated imaging. Accelerated imaging is often used to reduce scan time [39]. One of the benefits of accelerated imaging is increased patient comfort and compliance, which leads to less motion artifacts and may decrease the risk of study exclusion due to unusable imaging [30]. On the other hand, accelerated (parallel) imaging generally has a lower signal-to-noise ratio and may suffer from residual aliasing artifacts, altering tissue contrasts, as well as noise enhancements [39]. Depending on the underlying approach for defacing as well as brain atrophy estimation, these differences between unaccelerated and accelerated imaging may be further pronounced by local and distant effects of linear or nonlinear registration of an image volume to a template or by using information from the image background in order to classify tissue (e.g., in a Bayesian approach). Consequently, studies with different approaches to atrophy estimation have shown varying degrees of differences in atrophy assessments when using accelerated vs. unaccelerated imaging [28–31]. Likely, for the same reasons, defacing of unaccelerated imaging generally was more robust, with the exception of PyDeface and to some degree mri_reface, which performed well on both accelerated and unaccelerated imaging.

In synopsis, choosing the most suitable approach for defacing is a multifactorial decision. For example, fsl_deface may currently provide a better defense than PyDeface against identification of a subject [3], but results of brain atrophy estimation may be biased in the frontal and frontobasal brain—especially in accelerated imaging. Further research is needed for different analysis pipelines and defacing approaches to carefully consider the trade-offs in result accuracy and privacy to choose the most suitable approach for the task at hand.

Our study is limited by restricting the analysis to only include AD patients from the ADNI database, and therefore, our results may not be transferable to other cohorts. However, ADNI was deliberately chosen, since it offers raw, unprocessed, non-defaced DICOM data of a wide variation of examinations from a large number of scanners from different vendors, which is expected to make our results more generalizable. Lastly, it has to be noted that different software versions of the defacing approaches or CAT12/SPM12 might lead to different results, and the respective release notes need to be monitored for major changes.

## Conclusion

Given the recent successes of applying face recognition algorithms to T1 imaging of the brain, some form of de-identification of MRI scans depicting facial features or the ears must be strongly considered when making data publicly available and possibly even when sending data to, e.g., cloud-based processing or analysis services. Especially PyDeface showed very good results with negligible impact on atrophy estimation. mri_reface was found to be very promising and future versions should be re-evaluated. Furthermore, veganbagel demonstrated robust atrophy estimation results when comparing initial and repeat full face, unmodified imaging.

## Data Availability

The datasets analyzed during the current study are available in the Alzheimer's Disease Neuroimaging Initiative (ADNI) repository, http://adni.loni.usc.edu. veganbagel is available from https://github.com/BrainImAccs/veganbagel. afni_refacer is available from https://afni.nimh.nih.gov. fsl_deface is available from https://fsl.fmrib.ox.ac.uk. mri_deface is available from https://surfer.nmr.mgh.harvard.edu/fswiki/mri_deface. mri_reface is available from https://www.nitrc.org/projects/mri_reface. PyDeface is available from https://github.com/poldracklab/pydeface. spm_deface is available from https://www.fil.ion.ucl.ac.uk/spm/.

## Abbreviations

AD: Alzheimer's disease
ADNI: Alzheimer's Disease Neuroimaging Initiative
CAT: Computational anatomy toolbox
IQR: Interquartile range
MRI: Magnetic resonance imaging
QC: Quality control
RMSE: Root-mean-square error
SD: Standard deviation
SPM: Statistical parametric mapping
Veganbagel: Volumetric estimation of gross atrophy and brain age longitudinally
